# Regulatory effects of a Mnk2-eIF4E feedback loop during mTORC1 targeting of human medulloblastoma cells

**DOI:** 10.18632/oncotarget.2319

**Published:** 2014-08-06

**Authors:** Frank Eckerdt, Elspeth Beauchamp, Jonathan Bell, Asneha Iqbal, Bing Su, Rikiro Fukunaga, Rishi R. Lulla, Stewart Goldman, Leonidas C. Platanias

**Affiliations:** ^1^ Robert H. Lurie Comprehensive Cancer Center and Division of Hematology- Oncology, Feinberg School of Medicine, Northwestern University, Chicago, IL,USA; ^2^ Department of Medicine, Jesse Brown VA Medical Center, Chicago, IL, USA; ^3^ Division of Hematology and Oncology, Ann & Robert H. Lurie Children's Hospital of Chicago, Chicago, IL, USA; ^4^ Department of Immunobiology, Yale University School of Medicine, New Haven, CT, USA; ^5^ Shanghai Institute of Immunology Department of Microbiology and Immunology, Shanghai JiaoTong University School of Medicine, Shanghai, China; ^6^ Laboratory of Biochemistry, Osaka University of Pharmaceutical Sciences, Osaka, Japan

**Keywords:** TOR, Mnk, rapamycin, medulloblastoma

## Abstract

The mTOR pathway controls mRNA translation of mitogenic proteins and is a central regulator of metabolism in malignant cells. Development of malignant cell resistance is a limiting factor to the effects of mTOR inhibitors, but the mechanisms accounting for such resistance are not well understood. We provide evidence that mTORC1 inhibition by rapamycin results in engagement of a negative feedback regulatory loop in malignant medulloblastoma cells, involving phosphorylation of the eukaryotic translation-initiation factor eIF4E. This eIF4E phosphorylation is Mnk2- mediated, but Mnk1-independent, and acts as a survival mechanism for medulloblastoma cells. Pharmacological targeting of Mnk1/2 or siRNA-mediated knockdown of Mnk2 sensitizes medulloblastoma cells to mTOR inhibition and promotes suppression of malignant cell proliferation and anchorage-independent growth. Altogether, these findings provide evidence for the existence of a Mnk2-controlled feedback loop in medulloblastoma cells that accounts for resistance to mTOR inhibitors, and raise the potential for combination treatments of mTOR and Mnk inhibitors for the treatment of medulloblastoma.

## INTRODUCTION

Medulloblastoma is the most common malignant brain tumor in children, comprising over 20% of all central nervous system tumors [1]. Recent advances in genomic medicine have led to the identification of four subtypes of medulloblastoma based on the presence of distinct genetic markers [2-4]. These include: WNT, sonic hedgehog (SHH), group 3, and group 4 (reviewed in [5]). The identification of distinct medulloblastoma subgroups has suggested that specific targeting of dysregulated pathways should have significant therapeutic implications. However, a frequent challenge for targeted intervention is the emergence of resistance to therapy because of compensatory activation of alternative signaling pathways. Thus, it is likely that effective targeted therapies for medulloblastoma will require combined regimens, aimed to simultaneously target resistance mechanisms. For instance, mouse models involving medulloblastomas with mutations in the sonic hedgehog (Shh) pathway frequently develop resistance to Smoothened (Smo) antagonists over time [6]. However, when NVP-BEZ235, an inhibitor of mammalian target of rapamycin (mTOR) and PI3K is used in combination with the smoothened antagonists, resistance is substantially delayed or even prevented [6]. This suggests that inhibitors of the PI3K/mTOR pathway may be promising in combination therapies for certain medulloblastoma subgroups.

The mTOR pathway mainly regulates initiation of mRNA translation, ultimately controlling protein synthesis and expression [7, 8]. It is well established that the overall rate of protein synthesis is an important determinant of cancer cell metabolism [9]. Many previous observations have indicated that dysregulated growth pathways in human cancers are involved in the control of translation supporting cell proliferation and survival [8]. In response to nutrients and growth factors, activation of Akt/mTOR pathways results in enhanced global protein synthesis [8]. Among the key effectors of the mTOR pathway are the S6 kinase (p70-S6K1) and the eukaryotic translation initiation factor 4E-binding protein 1 (4E-BP1), which acts as a translational repressor by inhibiting the function of the cap-binding protein, eukaryotic translation initiation factor 4E (eIF4E) [10].

MAP (mitogen-activated protein) kinase-interacting kinases (Mnk1/2) have been previously shown to phosphorylate the eukaryotic translation initiation factor 4E (eIF4E) on Ser-209 [11]. The phosphorylation of eIF4E on Ser-209 is frequently increased in cancer cells and eIF4E expression levels are upregulated in many tumors [12-14]. Phosphorylation of eIF4E on Ser-209 is crucial for its transforming/oncogenic ability [15, 16]. There is some evidence that phosphorylation of eIF4E at this site may account in part for resistance to mTORC1 inhibition [17-20], but the precise mechanisms remain to be defined. In the present study, we sought to determine whether eIF4E phosphorylation accounts for resistance to mTORC1 inhibition in medulloblastoma cells and, if so, to investigate the mechanisms of cross-talk between the mTOR and Mnk pathways in medulloblastoma cells. Our data provide evidence for a unique Mnk2-mediated mechanism and suggest that combinations of mTOR and Mnk2 inhibitors may provide a therapeutic approach for human medulloblastoma.

## RESULTS

**Figure 1:**
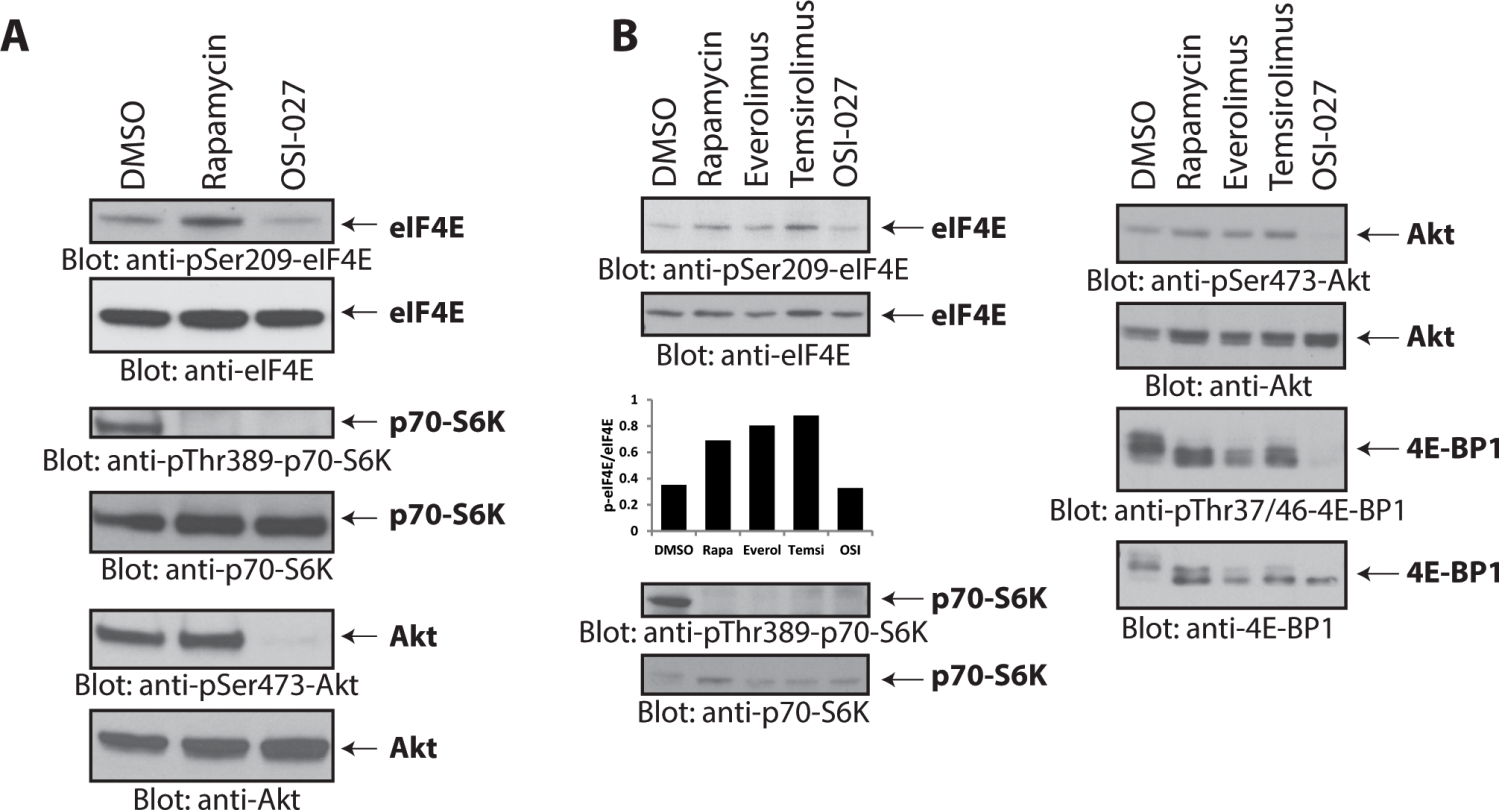
Rapalogs increase phosphorylation of eIF4E on Ser-209 in medulloblastoma cells (A) Daoy cells were incubated with rapamycin (20 nM) or OSI-027 (5 μM) for 90 minutes. Cell lysates were resolved by SDS-PAGE and immunoblotted with antibodies against the phosphorylated forms of eIF4E (pSer-209), p70-S6K (pThr-389), and Akt (pSer-473). The corresponding same blots were stripped and reprobed with antibodies against eIF4E, p70-S6K and Akt, respectively, as indicated. (B) Daoy cells were incubated with rapamycin (20 nM), everolimus (200 nM), temsirolimus (5 μM) or OSI-027 (5 μM) for 90 min. Cell lysates were resolved by SDS-PAGE and immunoblotted with antibodies against the phosphorylated forms of eIF4E (pSer-209), p70-S6K (pThr-389), Akt (pSer-473) and 4E-BP1 (pThr-37/46). The corresponding same blots were stripped and reprobed with antibodies against total eIF4E, p70-S6K, Akt and 4E-BP1, respectively as indicated. The bands from the anti-phospho-eIF4E and anti-eIF4E blots were quantitated by densitometry using ImageJ, and data were expressed as ratios of p-eIF4E/eIF4E, as shown in the bar graphs below the top 2 blots.

In initial studies, we sought to examine the effects of different mTOR inhibitors on the phosphorylation/activation of eIF4E in human medulloblastoma cells. As shown in Fig. 1, treatment of Daoy medulloblastoma cells with rapamycin induced phosphorylation of eIF4E on Ser-209 (Fig. 1A), suggesting activation of a feedback loop during mTORC1 inhibition in these cells. On the other hand, treatment with the ATP-competitive mTOR inhibitor OSI-027 did not result in an increase eIF4E phosphorylation (Fig. 1A). The activities and specificities of both rapamycin and OSI-027 were confirmed by monitoring phosphorylation of the mTORC1 substrate p70-S6K on Thr-389 and the mTORC2-mediated phosphorylation of Akt on Ser-473 [21, 22]. In addition to rapamycin, treatment with the rapalogs everolimus and temsirolimus also increased phosphorylation of eIF4E on Ser-209 (Fig. 1B).

**Figure 2:**
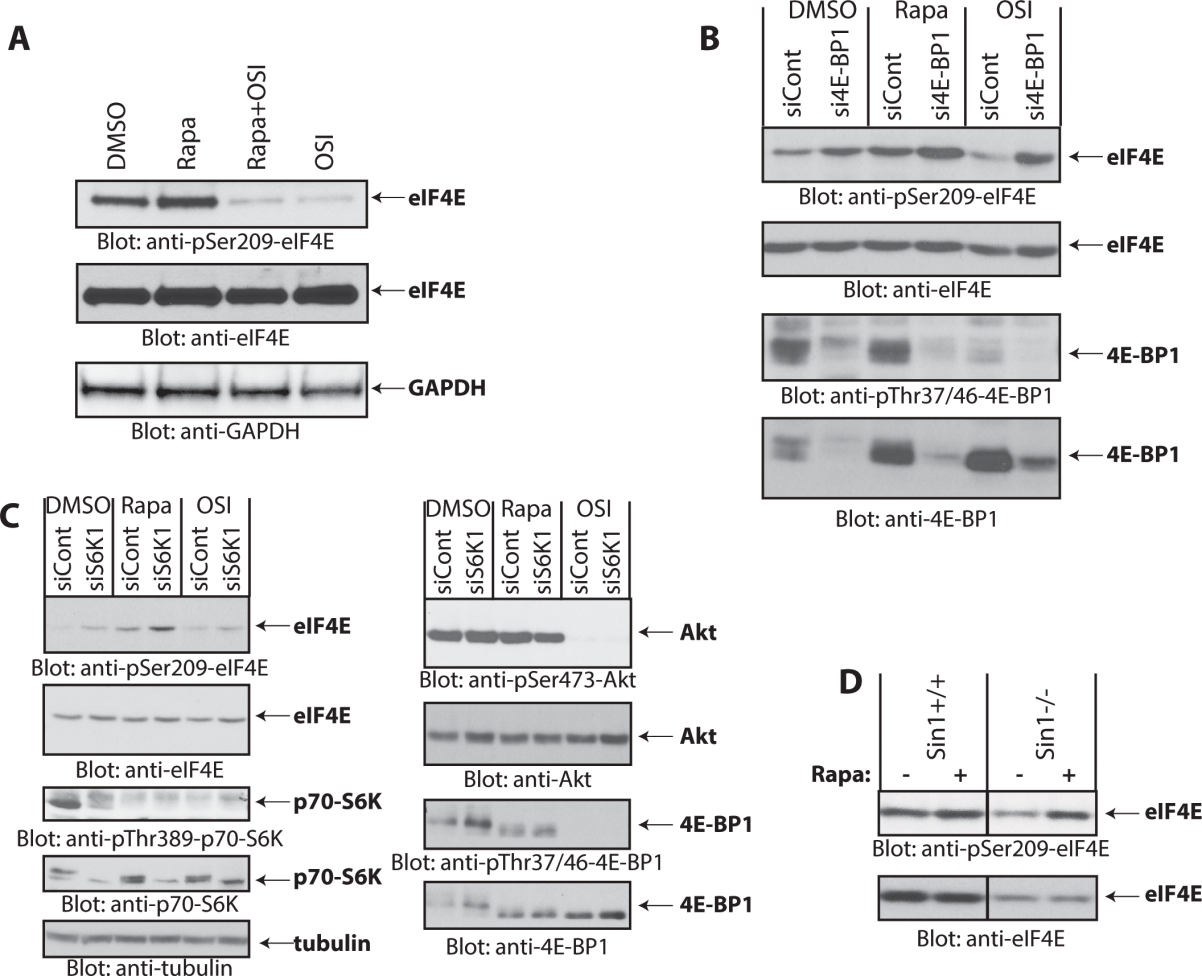
Rapamycin-induced phosphorylation of eIF4E (A) Daoy cells were incubated with rapamycin (25 nM) and/or OSI-027 (10 μM) for 90 min. Cell lysates were resolved by SDS-PAGE and immunoblotted with antibodies against the phosphorylated form of eIF4E (pSer-209) or GAPDH (*upper and lower panels*). Equal amounts of cell lysates from the same experiment were analyzed in parallel by SDS-PAGE and immunoblotted against eIF4E (*middle panel*). (B) Daoy cells were transfected with control or 4E-BP1 siRNA. After 48 hours, cells were treated with rapamycin (20 nM) or OSI-027 (5 μM) for 90 min. Cell lysates were resolved by SDS-PAGE and immunoblotted with antibodies against the phosphorylated forms of eIF4E (pSer-209) or 4E-BP1 (pThr-37/46). The same blot was stripped and reprobed with antibodies against eIF4E or 4E-BP1, as indicated. (C) Daoy cells were transfected with either control or p70-S6K1 siRNA. After 48 hours, cells were treated with rapamycin (20 nM) or OSI-027 (5 μM) for 90 min. Cell lysates were resolved by SDS-PAGE and immunoblotted with antibodies against the phosphorylated forms of eIF4E (pSer-209), p70-S6K (pThr-389), Akt (pSer-473), 4E-BP1 (pThr-37/46) or tubulin. The corresponding same blots were stripped and reprobed with antibodies against total eIF4E, p70-S6K, Akt or 4E-BP1, as indicated. (D) Sin1^+/+^ and Sin1^-/-^ MEFs were treated with Rapamycin (20 nM) for 90 min and equal amounts of protein were processed for immunoblotting with antibodies for phosphorylated eIF4E (pSer-209) (*upper panel*). The immunoblot with antibodies against total eIF4E protein was from lysates from the same experiment analyzed in parallel by SDS-PAGE (*lower panel*).

Rapalogs bind the FK506-binding protein (FKBP12) and this complex inhibits rapamycin-sensitive mTORC1 but not rapamycin-insensitive mTORC1 or mTORC2 complexes [8, 21, 23]. As OSI-027 did not induce phosphorylation of eIF4E on Ser209, we sought to investigate whether increased eIF4E phosphorylation is indirectly dependent on mTOR catalytic activity. Simultaneous treatment of Daoy cells with OSI-027 completely blocked rapamycin-induced eIF4E phosphorylation (Fig. 2A), suggesting that the rapamycin-induced increase of eIF4E phosphorylation on Ser-209 requires mTOR kinase activity. These findings also suggested that the induction of this mTOR kinase activity either involves mTORC2-mediated signaling events, or rapamycin-insensitive (RI) mTORC1 events. Notably, phosphorylation of 4E-BP1 on Thr-37/46 has been previously shown to be mediated by rapamycin-insensitive mTORC1 complexes in different malignant cell types [8, 21, 23, 24]. Therefore, we examined whether 4E-BP1 is required for rapamycin-mediated increase in eIF4E phosphorylation. As expected, siRNA-mediated knockdown of 4E-BP1 led to increased eIF4E phosphorylation (Fig. 2B, compare lanes 1 and 2), reflecting an increase in the amount of free eIF4E available for Mnk-mediated phosphorylation. However, treatment with rapamycin increased eIF4E phosphorylation further (Fig. 2B, compare lanes 2 and 4), suggesting that rapalog-induced eIF4E phosphorylation is independent of 4E-BP1 levels.

Inhibition of the mTORC1-p70-S6K pathway by rapalogs has been shown to relieve repression of a negative feedback loop, resulting in mTORC2 activation by preventing p70-S6K mediated Rictor phosphorylation on Thr-1135 [25, 26] and Sin1 phosphorylation on Thr-86 and Thr-398 [27]. As the catalytic mTOR inhibitor OSI-027 blocks rapalog-induced eIF4E phosphorylation, we examined the possibility that such phosphorylation is triggered by inhibition of the negative feedback-loop, resulting in activation of mTORC2 in response to p70-S6K inhibition. To this end, we knocked down p70-S6K using specific siRNA and investigated whether rapamycin is still able to induce eIF4E phosphorylation in the absence of p70-S6K. Knockdown of p70-S6K increased phosphorylation of eIF4E, but rapamycin treatment further increased eIF4E phosphorylation although p70-S6K levels were substantially reduced (Fig. 2C). This suggested that rapamycin-induced increase in eIF4E phosphorylation is independent of p70-S6K-regulated feedback loops. We also investigated whether rapamycin is capable of inducing eIF4E phosphorylation in the absence of Sin1, a crucial component of mTORC2 [28]. Using Sin1^-/-^ mouse embryonic fibroblasts (MEFs) [28, 29] we found that rapamycin still induced phosphorylation of eIF4E on Ser-209 in the absence of Sin1 (Fig. 2D). Altogether, these results indicated that the increase in eIF4E phosphorylation in response to rapamycin is independent of p70-S6K and Sin1/mTORC2 activity and is not dependent on inhibition of a conventional negative feedback loop.

**Figure 3:**
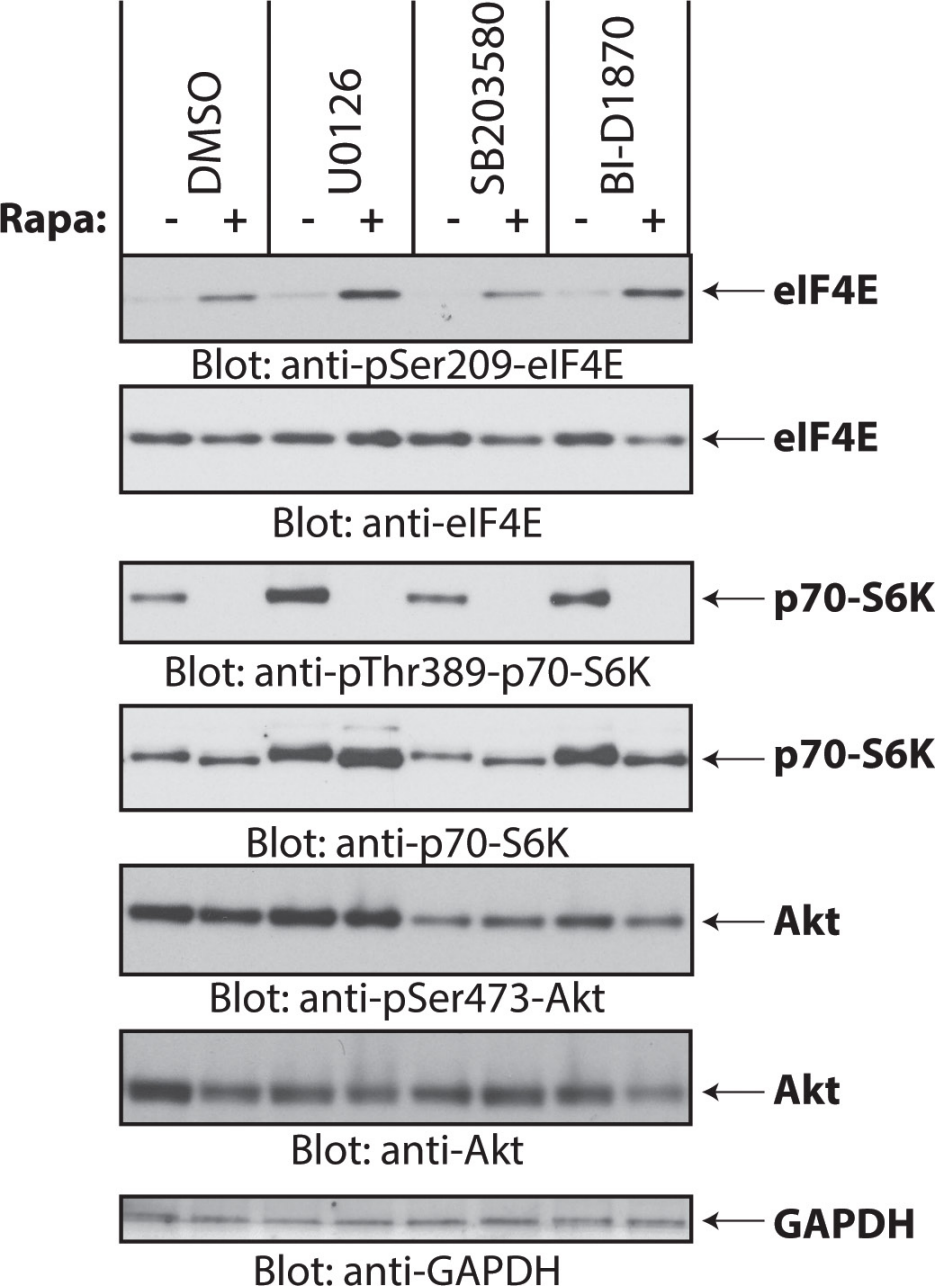
Rapamycin-induced phosphorylation of eIF4E is independent of the MAPK pathways Daoy cells were pretreated with DMSO or inhibitors for MEK (U0126, 10 μM), p38 (SB203580, 20 μM), Rsk1 (BI-D1870, 10 μM), for 60 min before addition of rapamycin (20 nM) for 2 hours. Equal amounts of protein were resolved by SDS-PAGE and processed by immunoblotting using antibodies for the phosphorylated forms of eIF4E (pSer-209), p70-S6K (pSer-389) and Akt (pSer-473). The blots were stripped and reprobed with antibodies for eIF4E, p70-S6K, Akt and GAPDH, as indicated.

**Figure 4:**
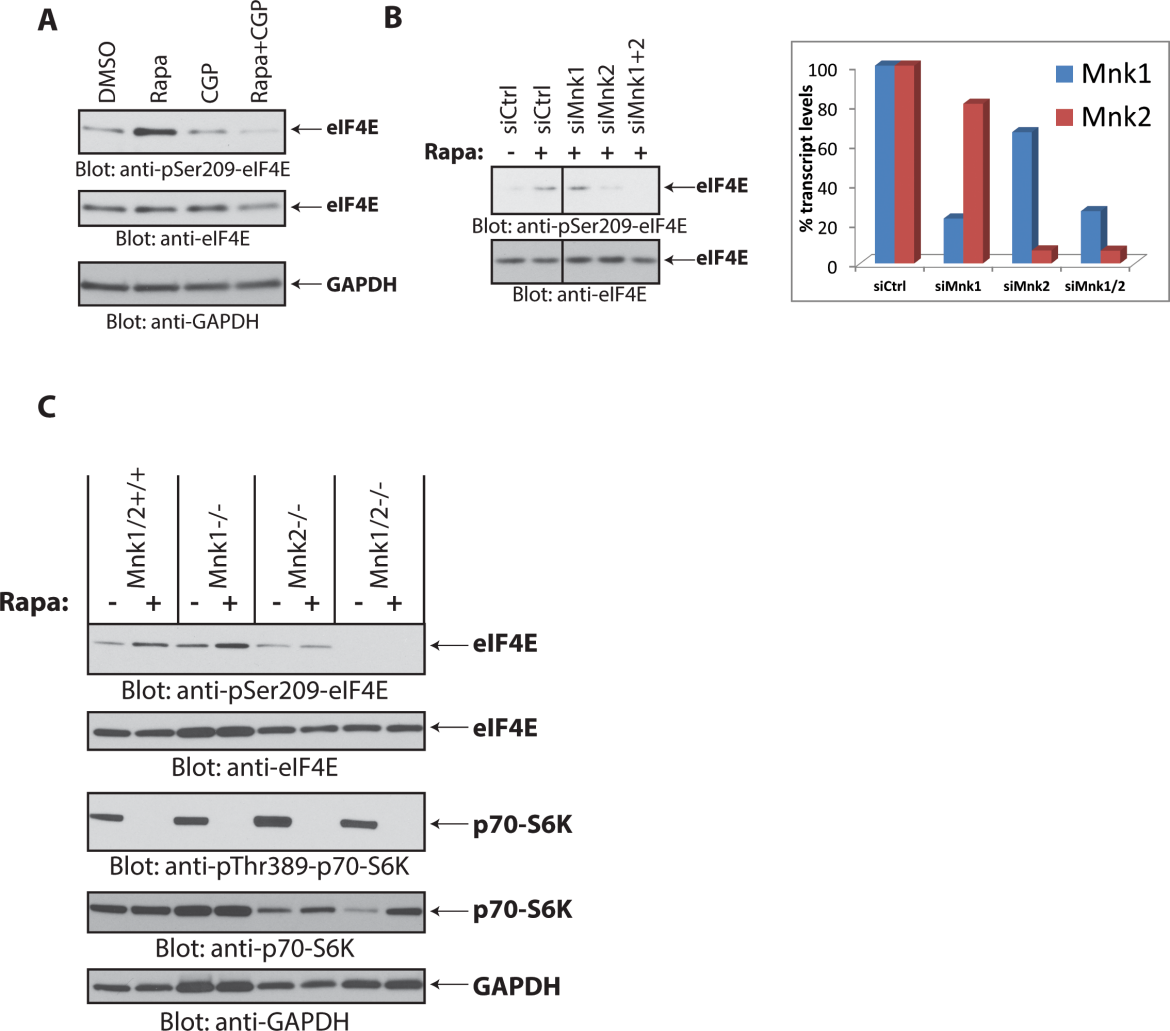
Mnk2 is required for rapamycin-induced eIF4E phosphorylation (A) Daoy cells were treated with rapamycin (20 nM) and/or CGP57380 (10 μM) for 90 min. Equal amounts of protein were processed by immunoblotting using antibodies for the phosphorylated form of eIF4E (pSer-209) or GAPDH. The membrane was stripped and reprobed with an antibody for eIF4E. (B) *(Left)* Daoy cells were transfected with control, Mnk1, Mnk2 and Mnk1+Mnk2 siRNAs. After 48 hours, cells were treated with rapamycin (20 nM) for 90 min, as indicated. Cell lysates were resolved by SDS-PAGE and immunoblotted with antibodies against the phosphorylated form of eIF4E (pSer-209). The same membrane was stripped and reprobed with an antibody for eIF4E. *(Right)* mRNA expression of Mnk1 and Mnk2 genes from cells transfected with the indicated siRNAs from the same experiment shown on the *left* panel, was assessed by quantitative RT-PCR in triplicates, using GAPDH for normalization. Data are expressed as percentages of control siRNA transfected cells. (C) Mnk1/2^+/+^, Mnk1^-/-^, Mnk2^-/-^ and Mnk1/2^-/-^ (DKO) MEFs were treated with rapamycin (20 nM) for 90 min. Equal amounts of protein were resolved by SDS-PAGE and immunoblotted with antibodies against phosphorylated eIF4E (pSer-209) or p70-S6K (pThr-389). Membranes were stripped and reprobed with antibodies for eIF4E, p70-S6K and GAPDH.

Previous work has shown that phosphorylation of eIF4E on Ser-209 is mediated by Mnk1 and Mnk2, which are effectors of the Erk1/2 and/or p38 MAPK pathways [30]. To define which components of the MAPK pathway are required for rapamycin-dependent eIF4E phosphorylation in medulloblastoma cells, we treated Daoy cells with a panel of protein kinase inhibitors to disrupt MAPK signaling and investigated induction of eIF4E phosphorylation under these conditions. Inhibition of MAPK pathway components by the MEK inhibitor U0126, the p38 MAPK inhibitor SB203580, or the Rsk1 inhibitor BI-D1870, failed to inhibit rapamycin-induced eIF4E phosphorylation (Fig. 3). This suggests that rapamycin induces eIF4E phosphorylation independently of MAPKs. As there is evidence that Mnk activity is required for rapamycin-induced eIF4E phosphorylation in leukemia cells [17, 18], we determined whether Mnks are required. Rapalog-dependent eIF4E phosphorylation was blocked by the Mnk inhibitor CGP57380 (Fig. 4A), reflecting a requirement for Mnk activity.

There has been previous evidence that MAPKs activate Mnk1 for inducible phosphorylation of eIF4E, whereas Mnk2 mainly contributes to eIF4E's basal, constitutive phosphorylation [31]. To define whether rapamycin-induced increase in eIF4E phosphorylation is mediated by Mnk1 or Mnk2, we knocked down Mnk1 or Mnk2 in Daoy medulloblastoma cells, and examined the effects of such knockdown on rapamycin-inducible eIF4E phosphorylation. Rapamycin treatment resulted in an increase in eIF4E phosphorylation in cells in which Mnk1 was knocked down, but not in cells with selective Mnk2 knockdown (Fig. 4B). These findings suggested that during treatment of medulloblastoma cells with rapamycin there is selective activation of Mnk2, but not Mnk1, for phosphorylation of eIF4E. Similar results were observed in Mnk knockout MEFs [31, 32], where rapamycin increased eIF4E phosphorylation in Mnk1^-/-^ MEFs, but failed to do so in Mnk2^-/-^ or Mnk1/2^-/-^ MEFs (Fig. 4C).

**Figure 5:**
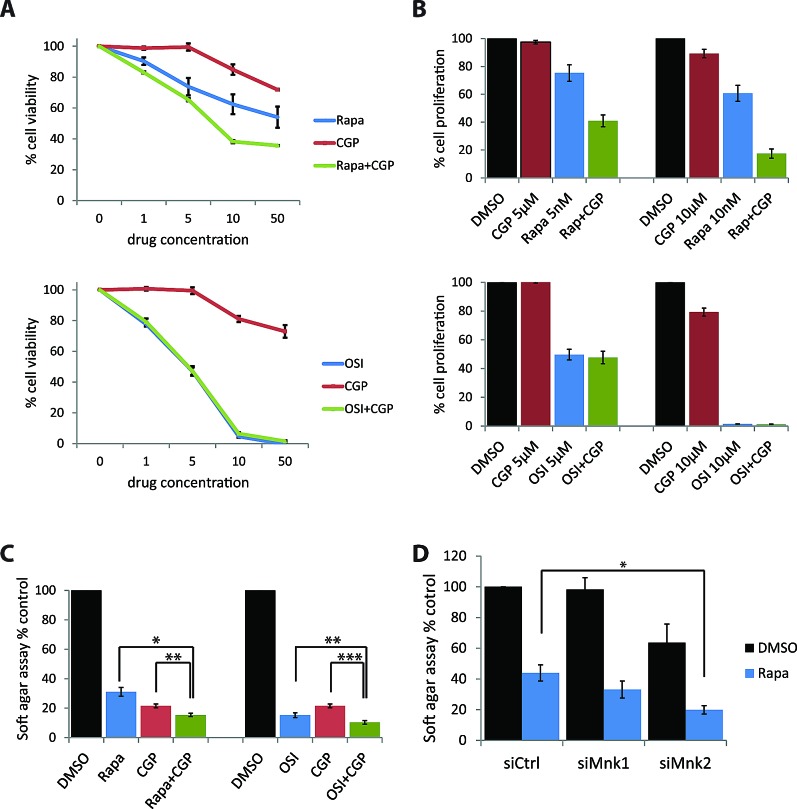
Simultaneous Mnk inhibition increases rapamycin-mediated inhibition of cell proliferation and colony formation (A) Daoy cells were incubated for five days with increasing concentrations of CGP57380 (1, 5, 10, 50 μM) in the presence or absence of increasing concentrations of rapamycin (1, 5, 10, 50 nM, upper panel) or OSI-027 (1, 5, 10, 50 μM, lower panel). Subsequently, cells were subjected to WST-1 proliferation assays. Means ± SE of the values from 3 independent experiments (each done in triplicates), are shown. Data are expressed as percentages of control DMSO treated samples. (B) Daoy cells were treated with the indicated concentrations of CGP57380, in the presence or absence of the indicated concentrations of rapamycin or OSI-027. After five days, cell numbers were counted using an automated cell counter. Means ± SE are shown as values of 3 independent experiments. Data are expressed as percentages of control DMSO treated samples. (C) Daoy cells were plated in soft-agar and treated with CGP57380 (10 μM) with or without rapamycin (10 nM) or OSI-027 (0.5 μM). After 7 days, colony formation was quantified using the fluorescent cell stain CyQUANT GR Dye (Cell Biolabs Inc.) in the Synergy HT Plate reader. Means ± SE of the values from 4 independent experiments are shown. Data are expressed as percentages of DMSO treated samples. *, p < 0.05, **, p < 0.005, ***, p <0.0005 using a paired t-test (D) Daoy cells were transfected with control, Mnk1, or Mnk2 siRNA. After 48 hours, cells were counted and equal numbers were plated in soft-agar and treated with DMSO as a control, rapamycin (10 nM) or OSI-027 (0.5 μM). Cells were incubated for 7 days. Colony formation was quantified using the fluorescent cell stain CyQUANT GR Dye (Cell Biolabs Inc.) in the Synergy HT Plate reader. Means ± SE are shown from 6 independent experiments. Data are expressed as percentages of DMSO treated control siRNA transfected samples. *, p < 0.05 using a paired t-test.

In subsequent studies, we sought to determine whether combined treatment of medulloblastoma cells with Mnk and mTOR inhibitors results in enhanced antineoplastic effects. Daoy cells were treated with the Mnk inhibitor CGP57380 and either rapamycin or OSI-027, and cells were subjected to cell viability assays. Increasing concentrations of CGP57380 alone only marginally inhibited cell proliferation in these cells (Fig. 5A). However, when CGP57380 was combined with increasing concentrations of rapamycin, it enhanced rapamycin's antiproliferative effect in a dose-dependent manner (Fig. 5A, upper panel). By contrast, CGP57380 failed to enhance the antiproliferative effects of increasing concentrations of OSI-027 (Fig. 5A, lower panel). Similar results were obtained when cell counts were used (Fig. 5B). Taken together, our results suggest that selective mTORC1 inhibition in medulloblastoma cells results in engagement of a Mnk2-dependent survival mechanism that can be counteracted by concomitant Mnk inhibition. In studies in which the effects of combination therapies on anchorage-independent growth of Daoy medulloblastoma cells were assessed, we found enhanced effects by the combinations of mTOR and Mnk inhibitors (Fig. 5C). Knockdown of Mnk2, but not Mnk1, using specific siRNAs enhanced rapamycin-dependent inhibition of anchorage-independent growth, as compared to rapamycin alone. (Fig. 5D).

**Figure 6:**
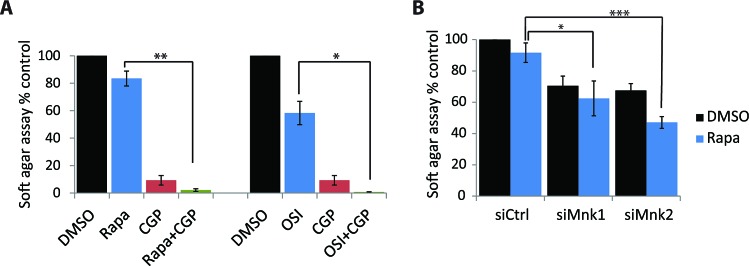
Inhibition of Mnk increases the inhibitory effects of mTOR inhibitors on D556 medulloblastoma cells (A) D556 cells were plated in soft-agar and treated with CGP57380 (10 μM) and incubated with or without rapamycin (20 nM) or OSI-027 (0.5 μM). Cells were incubated for 7 days. Colony formation was quantified using the fluorescent cell stain CyQUANT GR Dye (Cell Biolabs Inc.) in the Synergy HT Plate reader. Means ± SE are shown from 4 independent experiments. *, p < 0.05, **, p < 0.005 using a paired t-test (B) D556 cells were transfected with either control, Mnk1 or Mnk2 siRNA. After 48 hours, cells were counted and equal numbers were plated in soft-agar and treated with DMSO as control or rapamycin (10 nM). Cells were incubated for 7 days. Colony formation was quantified using the fluorescent cell stain CyQUANT GR Dye (Cell Biolabs Inc.) in the Synergy HT Plate reader. Means ± SE are shown from 4 independent experiments. Data are expressed as percentages of DMSO treated control siRNA transfected samples. *, p < 0.05, ***, p < 0.0005 using a paired t-test.

We also tested the effects of Mnk inhibition in another medulloblastoma cell line, D556. The combination of rapamycin with CGP57380 inhibited anchorage-independent malignant colony formation more than either drug alone (Fig. 6A). It should be noted that the D556 cell line was very sensitive to Mnk inhibition, as CGP57380 reduced colony formation to 9.2% (Fig. 6A), and knockdown of either Mnk1 or Mnk2 alone substantially reduced colony formation in D556 cells (siMnk1: 70.46%; siMnk2: 67.49%) (Fig. 6B). Both pharmacological inhibition of Mnks, or knockdown of Mnk1 or Mnk2, increased the sensitivity to rapamycin (Fig. 6). Knockdown of Mnk1 and Mnk2 in control (DMSO) cells resulted in similar inhibition of colony formation (Fig. 6B). Importantly, when combined with rapamycin treatment, knockdown of Mnk2, but not Mnk1, resulted in greatly impaired ability to form colonies in soft agar (Fig. 6B). Noteworthy, in both medulloblastoma cell lines (Daoy and D556) knockdown of Mnk2 had a stronger inhibitory effect on colony formation than knockdown of Mnk1. Taken together, these findings indicate that in both medulloblastoma lines, simultaneous inhibition of the mTOR and Mnk pathways results in substantially enhanced inhibition of anchorage-independent growth of malignant cells.

## DISCUSSION

There has been prior evidence that rapalogs exhibit antitumor effects against medulloblastoma cells, raising the potential for clinical use in the treatment of this malignancy [33, 34]. Here, we investigated the mechanisms by which rapamycin increases eIF4E phosphorylation in Daoy medulloblastoma cells and the relevance of this phosphorylation in counter-acting its antineoplastic effects. We found that rapalog-induced eIF4E phosphorylation is independent of 4E-BP1. We also found that rapamycin-induced eIF4E phosphorylation is not mediated by mTORC2 through negative feedback regulation, because it is independent of p70-S6K and Sin1/mTORC2. This finding is in agreement with a previous study demonstrating that rapamycin induces eIF4E phosphorylation independently of Rictor/mTORC2 in lung cancer cells [20].

Our studies also indicated that rapalog-induced eIF4E phosphorylation is mediated by Mnks independently of the canonical Mnk-activating pathway mediated by MAPKs [19]. While the exact mechanism by which rapamycin activates eIF4E in this context remains to be precisely defined, we found that this event is mediated by Mnk2 and not by Mnk1. It should be noted that another recent study employed a mass spectrometric approach and found that rapamycin alters phosphorylation of Mnk2 on Ser-437 in prostate cancer cells, triggering Mnk2 activation and eIF4E phosphorylation independently of MAPKs [35]. Our findings suggest a similar mechanism in medulloblastoma cells, as demonstrated by experiments demonstrating that Mnk2, but not Mnk1, is essential for rapamycin-induced eIF4E phosphorylation in Daoy cells.

The results of our studies raise the potential for concomitant targeting of Mnk2 as a means to enhance the antineoplastic effect of rapalogs in medulloblastoma. The Mnk inhibitor CGP57380 increased the antiproliferative effect of rapamycin, and significantly reduced colony formation in combination with either rapamycin or OSI-027. Molecular characterization of the Daoy cell line has revealed a global activation of Hh pathway genes including *Shh*, *Gli* family and *Ptch1*, as well as overexpression of *Bmi1* [36]. Thus, Daoy cells likely represent the SHH subgroup of medulloblastomas. To investigate whether the combination of mTOR and Mnk inhibition might be similarly effective in other medulloblastoma subgroups we extended our analysis by including D556 cells, which exhibit amplified *MYCC* [37]. As compared to Daoy cells, D556 cells were very sensitive to Mnk inhibition by both pharmacological inhibition and RNAi and, similar to Daoy cells, CGP57380 increased rapamycin's inhibitory effect on colony formation in D556 cells. Knockodwn of Mnk1 and Mnk2 reduced colony formation to similar levels in DMSO treated cells. However, in combination with rapamycin, knockdown of Mnk2 inhibited colony formation significantly more potently than knockdown of Mnk1, indicating a Mnk2 specific role in rapamycin activated negative feedback regulatory loops. Importantly, in both medulloblastoma cell lines (Shh-subgroup and subgroup 3) the targeted inhibition of Mnk2 potently increased the antineoplastic action of rapamycin, likely by preventing activation of the Mnk2-eIF4E survival pathway. Thus Mnk inhibition might be a promising anti-cancer strategy in these medulloblastoma subgroups. This finding is important because group 3 medulloblastomas have the worst prognosis of all four subgroups and new efficient targeted approaches are needed [38]. It should be noted that targeting the Mnk pathway represents an attractive target for the treatment of these cancers because Mnk activity – while being necessary for eIF4E-mediated oncogenic transformation – is dispensable for normal development [31].

## MATERIALS AND METHODS

### Cell Lines, Reagents and Antibodies

Daoy and D556 medulloblastoma cells were maintained in DMEM supplemented with 10% (v/v) fetal bovine serum and antibiotics at 37°C in 5% CO_2_. Immortalized Mnk1/2^+/+^, Mnk1^-/-^, Mnk2^-/-^, Mnk1/2^-/-^, Sin1^+/+^ and Sin1^-/-^ MEFs were grown in DMEM supplemented with 10% (v/v) fetal bovine serum and antibiotics as described previously [39, 40]. The antibodies against p-eIF4E (pSer-209), p-p70-S6K (pThr-389), p-Akt (pSer-437 and pThr-308), p-4E-BP1 (pThr-37/46), eIF4E, p70-S6K, Akt, 4E-BP1 were obtained from Cell Signaling Technology (Danvers, MA). The antibodies against alpha-tubulin were from Santa Cruz Biotechnology, Inc. (Santa Cruz, CA) and for GAPDH from Millipore (Billerica, MA). Rapamycin and Temsirolimus were from Sigma-Aldrich, Everolimus was from LC Laboratories, OSI-027 was from ChemieTek, CGP-57380 was from Santa Cruz Biotechnology Inc., BI-D1870 was from Symansis and U0126, SB203580 and LY294002 were from Calbiochem. For gene silencing of 4E-BP1 and p70-S6K1 by siRNA, cells were transfected with control non-targeting or double-stranded RNA oligonucleotides (Santa Cruz Biotechnology, Inc.) directed to 4E-BP1 (sc-29594) and p70-S6K1 (sc-36165). For gene silencing of Mnk1 and Mnk2 by siRNA, cells were transfected with control non-targeting or double-stranded RNA oligonucleotides (Dharmacon, Lafayette, CO) directed to Mnk1 (SMARTpool L-004879-00-0005) and Mnk2 (SMARTpool L-004908-0005). For transfection, Lipofectamine RNAiMAX Reagent (Invitrogen, Carlsbad, CA) was used, according to the manufacturer's instructions.

### Cell Lysis and Immunoblotting

Cells were treated, lysed in phosphorylation lysis buffer containing protease and phosphatase inhibitors, and prepared for immunoblotting as in our previous studies [29, 39].

### Cell Viability/Proliferation Assays

Experiments using the 3-(4,5-dimethyl-2-thiazolyl)-2,5-diphenyltetrazolium bromide (MTT) methodology were carried out using the Cell Proliferation Reagent (WST-1) assay kit (Roche, Mannheim, Germany) according to the manufacturer's instructions. In brief, for Daoy and D556 cells, 2000 cells per well were seeded in a 96-well plate and incubated with the indicated inhibitors. After 5 days, 10% (v/v) WST-1 reagent was added to each well and absorbance at 450nm was analyzed (using absorbance at 600nm as a reference wavelength), using an Epoch Plate reader and Gen5 software from BioTek Instruments Inc. For proliferation, cells were counted using an automated cell counter (Scepter, Millipore).

### Colony Formation Assay/Anchorage-Independent Cell Growth

For investigation of anchorage-independent cell growth soft-agar assays were performed using the CytoSelect 96-Well Cell Transformation Assay Kit (Cell Biolabs, Inc.) according to the manufacturer's instructions. In brief, 2500 cells per well were seeded in soft-agar in a 96-well plate and incubated at 37°C in 5% CO_2_ with the indicated inhibitors. For siRNA experiments, cells were transfected with the indicated siRNAs 2 days prior to seeding equal cell numbers into the 96-well format in soft-agar, followed by drug treatment. After 7 days, agar was solubilized and cells were lysed according to the manufacturer's instructions. Colony formation was quantified using the fluorescent cell stain CyQUANT GR Dye (Cell Biolabs Inc.) in the Synergy HT Plate reader using Gen5 software from BioTek Instruments Inc.

### Quantitative Real Time PCR

Cellular mRNA was reverse-transcribed into cDNA using the Omniscript TR kit and oligo(dT) primer (Qiagen) as in our previous studies [41]. Quantitative PCR using commercially available Taqman primers (Applied Biosystems) to determine *Mnk1* and *Mnk2* mRNA expression was used. GAPDH was used for normalization.
